# Therapeutic Effect of Intratympanic Injection of Dexamethasone plus Hyaluronic Acid on Patients with Meniere's disease 

**Published:** 2019-07

**Authors:** Mehrdad Rogha, Hamidreza Abtahi, Leila Asadpour, Hossein Ghazavi, Reza Pourmohammadi, Masoud Maleki, Keyvan Ghadimi

**Affiliations:** 1 *Department of Otorhinolaryngology, School of Medicine, Isfahan University of Medical Science, Isfahan, Iran.*; 2 *Department of Ophthalmology, Isfahan Eye Research Center, School of Medicine, Isfahan University of Medical Science, Isfahan, Iran.*; 3 *School of Medicine, Isfahan University of Medical Science, Isfahan, Iran.*

**Keywords:** Dexamethasone, Hyaluronic acid, Meniere’s disease

## Abstract

**Introduction:**

Intratympanic therapy, as a widely used treatment for inner ear diseases, is regarded as a therapeutic method in controlling the vertigo of the patients with Meniere’s disease. This study was designed to evaluate the effect of the Intratympanic dexamethasone-hyaluronic acid combination on patients suffering from Meniere’s disease.

**Materials and Methods:**

This study was a clinical trial on patients with Meniere’s disease during 2016-2017. Patients received two Intratympanic injections of dexamethasone plus hyaluronic acid as a mixture within a month. Before and 2 weeks after the intervention, pure tone average (PTA) at 0.5, 1, 2, and 4 KHz frequencies, speech discrimination score (SDS), dizziness handicap inventory (DHI), and tinnitus handicap inventory (THI) scores were evaluated for each patient. The obtained scores were statistically analyzed.

**Results:**

This study was conducted on a total number of 25 patients with Meniere's disease. The mean age of participants in this study was 44.71±4.92 years. Gender distribution among participants revealed that 36% of patients were male. The mean values of PTA, SDS, and THI were not significantly different before and after the intervention. However, the mean score of DHI decreased significantly after the intervention (P<0.001).

**Conclusion:**

Intratympanic dexamethasone/hyaluronic acid had a positive effect on the vertigo of the investigated patients without any significant improvement in hearing impairment and tinnitus in the short term.

## Introduction

Meniere’s disease (MD) is an inner ear disease, defined as the presence of recurrent spontaneous episodic vertigo accompanied by ipsilateral hearing loss, aural fullness, and tinnitus (1). The clinical diagnosis of Meniere’s disease is based on the presence of symptoms and the absence of the other causes of vertigo (1,2). Although the exact etiology of this disease is still unknown, the endolymphatic hydrops is considered as the main pathophysiological mechanism for the treatment of this disease (3). Since it is difficult to diagnose this disease at the early stages, an accurate determination of the prevalence and incidence of Meniere’s disease is hardly possible. However, the incidence of Meniere's disease is roughly 190 cases in every 100,000 of the population in the United States (4). 

There is no definite treatment for Meniere's disease. A variety of therapeutic modalities have been presented consisting of changes in nutritional diet, medicines, such as steroids or Gentamicin, and conservative or ablative surgeries (5). Despite the beneficial effect of oral medications and modifications in the lifestyle of such patients, there is a large number of participants suffering from a severe type of Meniere's disease without acceptable improvement (3,6). Nowadays, Intratympanic (IT) routes of therapy are extensively used in the treatment of inner ear diseases, and is also popular in controlling vertigo in patients with Meniere's disease (7). As a therapeutic modality, Intratympanic steroid injection was presented 15 years ago to treat some patients with inner ear disease, and since then different studies have been carried out to evaluate the therapeutic results (8). A study on 11 patients with Meniere's disease evaluated the effects of Intratympanic dexamethasone on controlling the patient's vertigo, which reported the success of this type of treatment (9). Another retrograde evaluation of Intratympanic dexamethasone was indicative of controlling vertigo in 91% of Meniere's disease patients after one course of injection (7). 

Hyaluronic acid is a viscous, high-molecular-weight polysaccharide that cannot penetrate the round window membrane and has osmotic effects on perilymph (10). Combining dexamethasone and hyaluronic acid may elongate the contact time of dexamethasone with round window membrane, which in turn increase inner ear corticosteroid absorption. A study by Gouveris et al. assessed the effects of the Intratympanic injection of dexamethasone plus hyaluronic acid on patients with sudden sensory neural hearing loss. The obtained results revealed significant hearing improvement in these patients who were resistant to intravenous steroid therapy (11). Moreover, another study compared the effects of intravenous steroids with and without Intratympanic injection of steroids with hyaluronic acid. The findings suggested that there was no significant difference between these two groups (12).

Due to the popularity of Intratympanic steroid therapies in patients with inner ear diseases and recent use of Intratympanic steroid with hyaluronic acid in patients with sudden sensory neural hearing loss, this study aimed to evaluate the effect of Intratympanic dexamethasone plus hyaluronic acid on patients with Meniere’s disease. 

## Materials and Methods

This study was a clinical trial on patients with Meniere's disease referring to Alzahra and Kashani hospitals of Isfahan, Isfahan, Iran, during 2016-2017. The inclusion criteria in this study were patients in the definite category of Meniere's disease (based on American committee of Otolaryngology-Head & neck surgery guideline) (13), and patient’s resistant to other treatments, such as lifestyle modifications, vasodilators or diuretics during the last 6 months. The exclusion criteria included patients with the history of otologic surgery, other pathologies in the middle and inner ear, and unwillingness to participate in the research project.

To obtain patients’ medical information, all the participants were interviewed before the injections. Next, they underwent ENT otologic evaluations, including otoscopy, tuning fork tests, Romberg and Unterberger/Fukuda stepping tests, evaluating nystagmus, audiometry, and tympanometry.

The diagnosis of Meniere's disease was determined clinically based on the history of episodic vertigo, aural fullness, tinnitus, and the presence of sensory neural hearing loss. In addition, all patients were subjected to magnetic resonance imaging to identify retro cochlear pathologies. All the patients received two intratympanic injections of dexamethasone (i.e., 0.3 and 8 mg/ml; manufactured by Abidi company, Iran) plus 0.2 ml of hyaluronic acid (0.2mg/ml; Hyalgan manufacture by Fidia Pharma USA). The interval between the two injections was one month. Patients were positioned supine with the head toward the unaffected side. Anesthesia was induced by a piece of gel foam soaked with 4% lidocaine solution on the lateral surface of the eardrum, left in place for 10 min. 

About 0.5 ml of the mixed solution was injected into the middle ear through an anterosuperior quadrant of the eardrum by a 0.6 mm needle. The patients were trained to keep their head in the same position for about 20 min after the injections to maximize the contact of medication and round window membrane.

Before and 2 weeks after the intervention, hearing impairment was assessed by pure tone average (PTA) at 0.5, 1, 2, and 4 KHz frequencies and speech discrimination score (SDS). Vertigo was evaluated through dizziness handicap inventory (DHI), a 2-item self-reported questionnaire that assesses the impact of vertigo on daily life utilizing self-perceived handicap measurement (14). To assess tinnitus, Tinnitus Handicap Inventory (THI) was employed. It is a self-administered test that determines the degree of distress suffered by the patient (15). All the tests were taken when patients were not in the episodes of Meniere's disease.

The collected data were analyzed using SPSS (version 20; SPSS crop., Chicago, IL, USA). For the quantitative phase of the study, mean and standard deviation (SD) were reported. On the other hand, frequency and percentage were used for qualitative data analysis. The comparison of variables before and after the intervention was accomplished by the application of paired t-test. P-value less than 0.05 was considered statistically significant. 

Regarding the ethical considerations, this study was registered in the Iranian Registry of Clinical Trial (IRCT20130311012782N25). Moreover, it was approved by the Regional Bioethics Committee of  International Union of Microbiological Societies.

## Results

**Fig 1:**
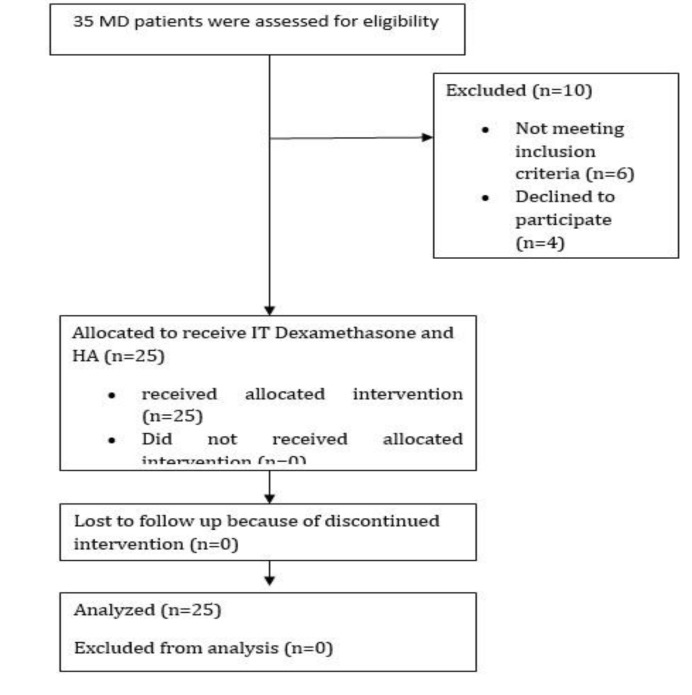
Trial profile of patients with Meniere's disease who received Intratympanic injection of dexamethasone plus hyaluronic acid

Among 35 patients who were assessed at the beginning, 25 patients were eligible to enter the study. All 25 patients remained at the end of the study, and no one was excluded from the study. The mean age of participants was 44.71±4.92 years within the age range of 34-52 years. Regarding gender distribution, 36% (n=9) of the subjects were male (Fig.1) (Table.1).

**Table 1:** Demographic variables of the investigated participants

**variable**		**Mean (SD) or Number (%)**
Age		44.71 (4.92)
Gender	Male	9 (36)
	female	16 (64)

**Fig 2:**
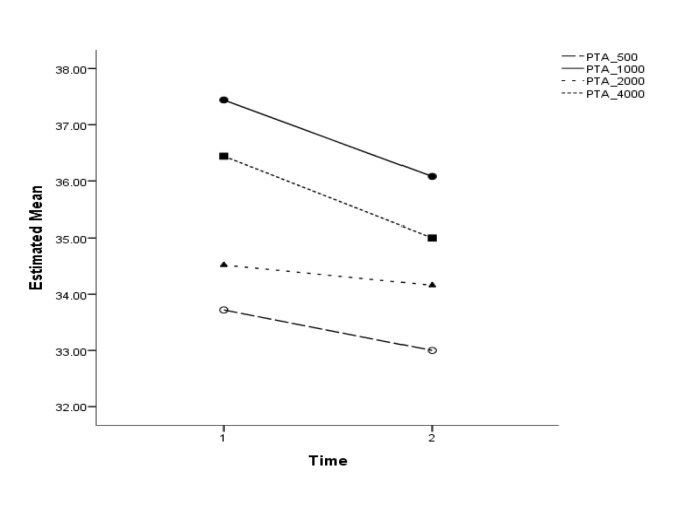
PTA changes in different frequencies in patients with Meniere's disease

**Fig 3:**
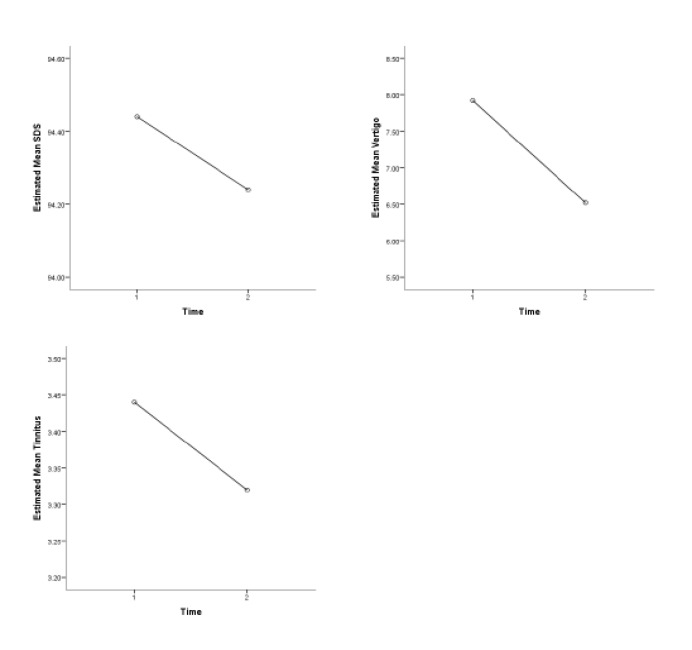
SDS, THI and DHI changes in patients with Meniere's disease

As can be seen in Figure 2, the mean scores of PTA were not significantly different at 0.5, 1, 2, and 4 KHz frequencies before and after the intervention (P=0.19, 0.06, 0.59, and 0.06, respectively). The mean values of SDS were roughly similar before and after the intervention, meaning that the investigated intervention led to no significant outcomes (P=0.09). Regarding the evaluation of vertigo, the mean scores of the DHI questionnaire were 7.92 and 6.52 before and after the intervention, respectively (P<0.001). Considering the analysis of tinnitus, The mean score of THI questionnaire for evaluating tinnitus was 3.44 before intervention and 3.22 after the intervention, which revealed no significant difference (P=0.08) (Table.2) (Fig.3).

**Table 2:** Mean values of pure tone average, speech discrimination score, dizziness handicap inventory, and tinnitus handicap inventory scores for Meniere's disease patients before and after the intervention

**Variables**	**Before intervention** **(Mean)**	**After intervention** **(Mean)**	**MD**	**SDD**	**SE (MD)**	**95% CI for MD**		**p**
						**LB**	**UP**	
PTA 500	33.72	33	0.72	2.68	0.53	-0.38	1.82	0.193
PTA 1000	37.44	36.08	1.36	3.48	0.69	-0.07	2.79	0.063
PTA 2000	34.52	34.16	0.36	3.32	0.66	-1.01	1.73	0.594
PTA 4000	36.44	35	1.44	3.78	0.75	-0.12	3	0.069
SDS	94.44	94.24	0.20	0.57	0.11	-0.03	0.43	0.096
DHI	7.92	6.52	1.4	1	0.2	0.98	1.81	<0.001*
THI	3.44	3.32	0.12	0.33	0.06	-0.01	0.25	0.083

D: Difference, MD: Mean Difference, SDD: Standard Deviation Difference, SE: Standard Error, CI: Confidence Interval

LB: Lower Bound, UP: Upper Bound, PTA: pure tone average, SDS: speech discrimination score, DHI: dizziness handicap inventory, THI: tinnitus handicap inventory scores, P: P-value.

* The mean DHI score was significantly different in patients before and after intervention

## Discussion

This study evaluated the effects of Intratympanic dexamethasone plus hyaluronic acid on the hearing impairment, vertigo, and tinnitus of patients with Meniere's disease, he obtained results revealed that although this type of Intratympanic injection can improve vertigo, it has no significant effect on hearing impairment and tinnitus. 

The administration of Intratympanic steroid for the treatment of inner ear diseases has some advantages. First and foremost, since local anesthesia is used in this type of treatment, the induced pain can be well tolerated by patients. In addition, this treatment led to no adverse effects induced by systemic steroids, including immune system suppression, weight gain, osteoporosis, avascular necrosis of hip, mood swing and endocrine and skin changes (16). Furthermore, intratympanic steroid passes directly through round window membrane into inner ear. Moreover, this type of treatment was only beneficial for the affected ear without any impact on contralateral inner ear. Besides, the steroid concentration within inner ear via intratympanic injection would be higher than other routes of steroid prescription; and last but not least, and finally Intratympanic injection is cheaper than systemic injections (17). On the other hand, the disadvantages of intratympanic injection include temporary pain, vertigo induced by injections, and some rare complications, such as otitis media, otomycosis, mastoiditis, and tympanic membrane perforation (17).

This study revealed that intratympanic dexamethasone plus hyaluronic acid injection could remarkably improve vertigo in patients with Meniere’s disease. One similar study on 61 patients with Meniere’s disease was indicative of 80% improvement in patients with vertigo (18). Likewise, a study conducted by Shea et al. on 28 patients with Meniere’s disease showed that the intratympanic and intravenous injection of dexamethasone can improve vertigo in 96.4% of patients (19). Similarly, Selivanaova et al. evaluated the effects of intratympanic dexamethasone and hyaluronic acid on hearing and vertigo attacks of 21 patients with Meniere's disease. The findings suggested that about 71.42% of patients respond to the treatment and did not show any symptom of Meniere's disease over a two-year follow-up period. It should be kept in mind that patients’ symptoms were evaluated generally but not individually (2). 

Another clinical trial on patients with Meniere's disease compared the effects of intratympanic dexamethasone plus hyaluronic acid and hyaluronic acid plus saline. The obtained results revealed that there were no significant differences between the two groups regarding the improvement of vertigo, tinnitus, and hearing threshold (20). The therapeutic effects of intratympanic steroids on patient’s vertigo was assigned to its anti-inflammatory effects on the labyrinth and perhaps its counteraction to the immune disorders of inner ear malfunction (21,22). In vitro studies showed that intralabyrinthine corticosteroid may alter sodium and fluid transport activity and as a result may relieve vertigo (21). Various studies on the effects of intratympanic steroids have reported different findings. Although most of these studies have evaluated the effects of Intratympanic steroids, including dexamethasone, a few of them investigate the intratympanic injection of dexamethasone plus hyaluronic acid. The concentration of steroid within the inner ear is estimated to be 20% higher when it is used with hyaluronic acid, compared to the time when it is used alone. 

The combination of hyaluronic acid with dexamethasone in intratympanic injections can increase the exposure time of round window membrane to dexamethasone, and consequently increases perilymphatic steroid concentration (10). To evaluate the effects of Dexamethasone plus hyaluronic acid on patients with Meniere's disease in depth, more researches with larger sample sizes are needed. 

This study did not show significant therapeutic effects of intratympanic Dexamethasone plus hyaluronic acid on the hearing abilities of patients with Meniere's disease. There are previous studies that evaluated the effects of intratympanic dexamethasone on patients with idiopathic hearing loss after the failure of oral and parenteral steroid therapies, which indicated the significant effects of this type of treatment on hearing (11). Other studies on patients with Meniere's disease revealed that 43-68% of patients had experienced improvement in hearing after the administration of intratympanic dexamethasone (19,23). The findings of some studies were in agreement with the obtained results of the current study, meaning that intratympanic steroids did not affect the hearing ability of patients Meniere's disease (20, 24). Although in the current study we evaluated the effects of intratympanic injection of a combined mixture of dexamethasone plus hyaluronic acid 2 weeks after the intervention (short term), previous studies assessed patients over a two-year follow-up period. Perhaps short-term therapy is insufficient to improve the hearing threshold. More studies should be conducted to disclose the details of different aspects of this therapeutic modality in short- and long-term.

In a study by Memari and Hassannia, intratympanic dexamethasone could significantly improve the hearing loss and tinnitus in a patient with Meniere’s disease (25). However, intratympanic dexamethasone/ hyaluronic acid had no effect on hearing loss and vertigo in the current study. In a study conducted by Masoumi et al., intratympanic dexamethasone or methylprednisolone improved vertigo and hearing loss in patients with Meniere's disease, indicating that methylprednisolone was more effective on hearing loss than dexamethasone (26). In another study, intratympanic dexamethasone temporary improved vertigo in patients with Meniere’s disease (27).

The current study offers the advantage of intratympanic double drug assessment (dexamethasone and hyaluronic acid). Most studies in the literature evaluated the intratympanic injection of dexamethasone alone, whereas a combination of dexamethasone and hydronic acid was administered in this study. Another advantage of this study refers to the limited number of injections (only two injections) that patients had. In most previous studies, patients were subjected to more repetitive injections, compared to the number of injections in the current study. This study suffers from some limitations. Firstly, this study was conducted on a small number of subjects, which hardly makes it possible to extend the results to the general population. Besides, some other factors, including patient's lifestyle, diet, probable complications, and previously used medications, were not investigated in this study. These confounding variables need to be taken into account in future studies. 

## Conclusion

In conclusion, the intratympanic injection of dexamethasone plus hyaluronic acid has positive effects on the patients with Meniere's disease, more specifically on vertigo; however, this injection cannot significantly improve hearing impairment and tinnitus. 
